# Performance Evaluation and Analysis for Gravity Matching Aided Navigation

**DOI:** 10.3390/s17040769

**Published:** 2017-04-05

**Authors:** Lin Wu, Hubiao Wang, Hua Chai, Lu Zhang, Houtse Hsu, Yong Wang

**Affiliations:** 1 State Key Laboratory of Geodesy and Earth’s Dynamics, Institute of Geodesy and Geophysics, Chinese Academy of Sciences, Wuhan 430077, China; wulin1108@gmail.com (L.W.); wanghb@whigg.ac.cn (H.W.); hchai@whigg.ac.cn (H.C.); zhanglu315@mails.ucas.ac.cn (L.Z.); hsuh@whigg.ac.cn (H.H.); 2 University of Chinese Academy of Sciences, Beijing 100049, China

**Keywords:** gravity aided navigation, underwater navigation, gravity matching, performance evaluation, requirements analysis

## Abstract

Simulation tests were accomplished in this paper to evaluate the performance of gravity matching aided navigation (GMAN). Four essential factors were focused in this study to quantitatively evaluate the performance: gravity database (DB) resolution, fitting degree of gravity measurements, number of samples in matching, and gravity changes in the matching area. Marine gravity anomaly DB derived from satellite altimetry was employed. Actual dynamic gravimetry accuracy and operating conditions were referenced to design the simulation parameters. The results verified that the improvement of DB resolution, gravimetry accuracy, number of measurement samples, or gravity changes in the matching area generally led to higher positioning accuracies, while the effects of them were different and interrelated. Moreover, three typical positioning accuracy targets of GMAN were proposed, and the conditions to achieve these targets were concluded based on the analysis of several different system requirements. Finally, various approaches were provided to improve the positioning accuracy of GMAN.

## 1. Introduction

A stable, precise passive navigation system is absolutely essential for long-distance and endurance underwater sailing. Currently, inertial navigation systems (INSs) are widely equipped on autonomous underwater vehicles (AUVs) to establish an underwater navigation system. However, INS navigation errors would accumulate with time, so additional aided navigation methods are needed to correct the cumulative errors to satisfy the requirement of practical application. Traditional aided navigation methods, like Global Navigation Satellite System (GNSS), radar, and celestial navigation, need AUVs to float near the surface, which would compromise the covertness. By contrast, gravimetry is not emanative, not easily detected and interfered with, and gravity aided navigation is stable and passive, so it is appropriate for underwater aided navigation. Especially in the GNSS-denied underwater environments, gravity aided navigation provides passive, all-weather, and undeniable navigation information that few other methods can offer.

In the past decades, gravity/gravity gradient aided navigation has been developed for underwater passive navigation systems to limit the INS error accumulation [1,2,3,4,5,6]. Several methods were explored for gravity matching aided navigation (GMAN). The terrain contour matching (TERCOM) algorithm derived from terrain matching aided navigation have been modified and employed for gravity matching navigation nowadays [7,8,9,10]. A relative positions-constrained pattern matching (RPCM) method using relative position offsets from the INS was proposed for gravity aided AUV navigation and obtained better performance [11]. Kalman filters were designed to estimate the state parameters of gravity/gravity gradient/geomagnetic aided navigation system [12,13,14,15,16]. The iterated closest contour point (ICCP) algorithm was constructed for vehicle localization in gravity maps [17,18,19]. With a shipborne marine gravimeter and GNSS devices, a sea trial of gravity aided inertial navigation was performed on two trajectories in the South China Sea. A 1.92 nautical mile positioning accuracy was obtained with 34 hours’ navigation, while the position errors of the INS accumulated to more than 12 nautical miles. Based on the comparison and analysis of the results of the two trajectories, the fitting degree between the gravity map and measurements, the dispersion of gravity changes, and the mean of the navigation capability values on the trajectory were considered as three of the key factors which most influence the positioning performance [20]. Additionally, simulation tests for gravity gradient-referenced aircraft navigation were conducted to verify the effects of various factors, such as database and sensor errors, flight altitude, initial errors, and update rates, on the navigation performance. Based on the results, requirements for gravity gradient-referenced navigation were established for certain positioning accuracies [21,22,23]. Additionally, gravimeters and gradiometers were adopted to compensate the INS [24,25]. Gravity and gravity gradient were used for underwater object detection, which is a significant subject in underwater navigation [26,27,28]. Unfortunately, few sea trials and performance evaluations of GMAN were reported in the literature. Thus, the conditions or requirements to achieve better accuracies with GMAN are still currently indistinct and unclear. Considering the rapid development of GMAN recently, the performance evaluation and analysis are particularly significant and meaningful for future marine experiments, system design, and implementation.

In this paper, simulation tests were performed for evaluating the performance of GMAN under the influence of various factors, such as database (DB) resolution, fitting degree of gravity measurements, number of samples in matching, and gravity changes in the matching areas. Gridded gravity anomaly maps which were calculated from satellite altimetry were adopted in the tests. Actual marine gravimetry was taken as a reference to design the simulation parameters. The results of average position errors were compared and analyzed. Finally, the GMAN system requirements for achieving some typical accuracy targets were proposed on the basis of simulation performance and evaluation.

## 2. Gravity Matching Aided Navigation

Generally, a GMAN system consists of an INS, the digital gridded gravity DB, which includes the gravity anomaly or gravity gradient values and their corresponding positions, a marine gravimeter, and navigation computers with navigation algorithms. The gravity DB was stored in computers in advance while sailing paths were also planned before the AUV missions. As the AUV moved, navigation information, including positions, was indicated by the INS and, simultaneously, gravity values were measured by the marine gravimeter.

**Figure 1:**
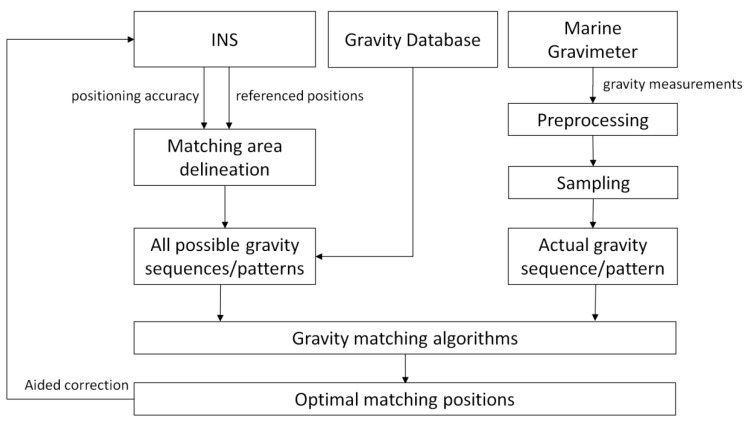
The flowchart of gravity matching aided navigation (GMAN).

After a passage the gravity measurements over a period of time were collected. Preprocessing, including several corrections, calibrations, and upward continuation, must be done to remove the influence of the Eötvös effect, temperature, sea conditions, instruments, and dive depth, etc. The matching area was delineated by INS indicated positions and INS accuracy. Then, the constructed gravity measurement sequence/pattern and gravity values in the matching area which were derived from the DB were inputted into gravity matching algorithms so that the optimal matching positions can be obtained. The flowchart of principle and scheme of GMAN is presented in Figure 1.

It can be found that the performance of GMAN is impacted by several parts in the system, such as the INS, gravity DB, gravimeter, preprocessing of gravity measurements, matching areas, matching algorithms, and so on. Based on the previous research, four typical factors, as follows, were focused in this study to quantitatively evaluate the performance of the GMAN system.

1. DB resolution

Gravity background DB is one of the most basic and fundamental components in the GMAN system. The resolution and accuracy of the gravity DB are the most significant parameters. Theoretically, it can be said that positioning accuracy of GMAN is limited by the DB resolution to some degree. The best approach to measuring marine gravity is to mount a very precise gravimeter on a ship. Unfortunately this ship coverage of the oceans is very sparse. With the development of satellite altimetry, which is an equally precise approach compared with marine shipboard gravimetry, currently the most advanced marine gravity anomaly DB derived from satellite altimetry and shipborne surveys has 1′ × 1′ spatial resolution (1′ = 1 nautical mile = 1853 m), and approximately 2 mGal accuracy (1 mGal = 10^−5^ m/s^2^) [29].

2. Fitting degree (FID) of gravity measurements

The FID of gravity measurements represents the root mean square (RMS) difference between measurements and gravity values at corresponding positions derived from DB. It is defined as:

(1) $$FID={\left(\frac{\sum_{i=1}^{n}\delta_{i}^{2}}{n}\right)}^{\frac{1}{2}}$$

where $\delta_{i}$ (*i* = 1, 2, …, *n*) is the sequence of gravity differences.

The gravity difference always exists since it comes from various sources, such as DB error, sensor error, measurement error, preprocessing error, and so on. Basically, the FID value is a reflection of measuring accuracy when certain DB and appropriate data processing methods are selected. The correlation calculations in navigation algorithms are directly affected by the FID value. In theory, a smaller FID value which means a better fitting degree, which usually leads to higher position accuracy. At present, most marine gravimeters around the world have a dynamic measuring accuracy ~1 mGal, the smallest FID values between altimeter-derived gravity DB and shipboard gravity range from about 1.6 to 3.6 mGal [29].

3. Number of measurement samples

For gravity matching algorithms, more gravity measurements in a sequence which contains more information for matching generally obtains a higher matching success rate and better positioning accuracy. Therefore, sailing paths through matching areas should be designed to ensure enough measurements can be collected. The Micro-g LaCoste marine gravimeters widely used around the world have a recording rate of 1 Hz [30].

4. Gravity changes in the matching areas

The features of gravity changes are adopted in the gravity matching algorithms. Better navigation results may be gained while gravity changes are much rougher in the matching area. Conversely, smooth gravity changes may lead to large errors and even invalid positioning results. A parameter named average gravity difference (AGD) was introduced here for the expression of the level of gravity changes in a certain area. It was defined as average gravity difference between neighbouring grids in DB or maps:

(2) $$AGD=\frac{\frac{\sum_{i=1}^{m}\sum_{j=1}^{n-1}\sigma_{ij}}{m\times \left(n-1\right)}+\frac{\sum_{i=1}^{n}\sum_{j=1}^{m-1}\omega_{ij}}{n\times \left(m-1\right)}}{2}$$

where $\sigma_{ij}$ (*i* = 1, 2, …, *m*; *j* = 1, 2, …, *n* − 1) and $\omega_{ij}$ (*i* = 1, 2, …, *n*; *j* = 1, 2, …, *m* − 1) are the gravity differences between neighbouring grids in *x* and *y* directions, respectively. With the overall survey of the global marine gravity anomaly DB, the AGD values can range from about 0.2 mGal/nautical mile to greater than 3.5 mGal/nautical mile in different areas of the oceans around the world.

These four factors are interdependent in that, together, they determine the performance of GMAN system. As a consequence, simulations and experiments need to be designed to test each of them.

## 3. Performance Evaluation of GMAN

Gravity maps located in different areas which derived from 1′ × 1′ resolution gridded marine gravity DB were chosen for the simulations.

**Figure 2:**
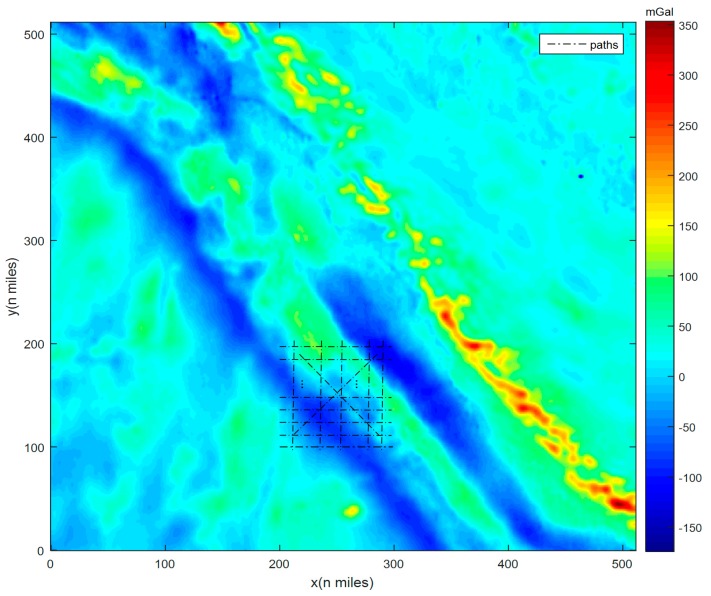
Paths cross the matching area in the gravity map.

The basic strategy is as follows: in each test, a set of 100 paths crossing the matching area were designed and simulated. An example is presented in Figure 2. It was assumed that the AUV would move along them. While the AUV was in motion, gravity measurements in an assigned matching length were collected and taken to match with the map. Thus, positioning results could be obtained at the end of each path. Consequently, the average position error could be calculated from the results of all 100 paths, corresponding to certain DB resolutions, FID values, numbers of samples, and AGD values in the area. Here, the gravity measurements were simulated from DB values added with noise, so the FID values can be calculated just with the noise. The matching length was determined by multiplying the number of measurement samples by the size of grid (resolution). Additionally, the correlation calculation methods and criterions used in matching algorithms were certainly the same to reduce the influence of them.

In matching algorithms the correlation coefficients between gravity map *P* and the gravity measurement sequence/pattern *S* would be calculated as follows:

(3) COEFS=COR(S,P)

where *COEFS* is the correlation coefficients which often be a matrix, and *COR* is the correlation algorithm. The mean square difference algorithm (MSD), which has been proven to be an effective and efficient correlation method, was chosen in our tests as the algorithm. It is defined as follows:

(4) $$MSD\left(x,y\right)=\frac{1}{M\times N}\overset{M-1}{\underset{u=0}{\sum }}\overset{N-1}{\underset{v=0}{\sum }}{\left[S\left(u,v\right)-P\left(x+u,y+v\right)\right]}^{2}$$

The minimum value of correlation coefficients *COEFS* identifies the matching position in the gravity map which is considered to be the optimal matching position.

After a series of tests, the average position errors from many different parameter settings were gathered and compared. Thus, the influence of each factor can be analyzed and evaluated.

### 3.1. DB Resolutions

Two schemes were designed to figure out how the DB resolutions influence the positioning performance of GMAN.

#### 3.1.1. Different Resolutions and Similar AGD Values

**Figure 3:**
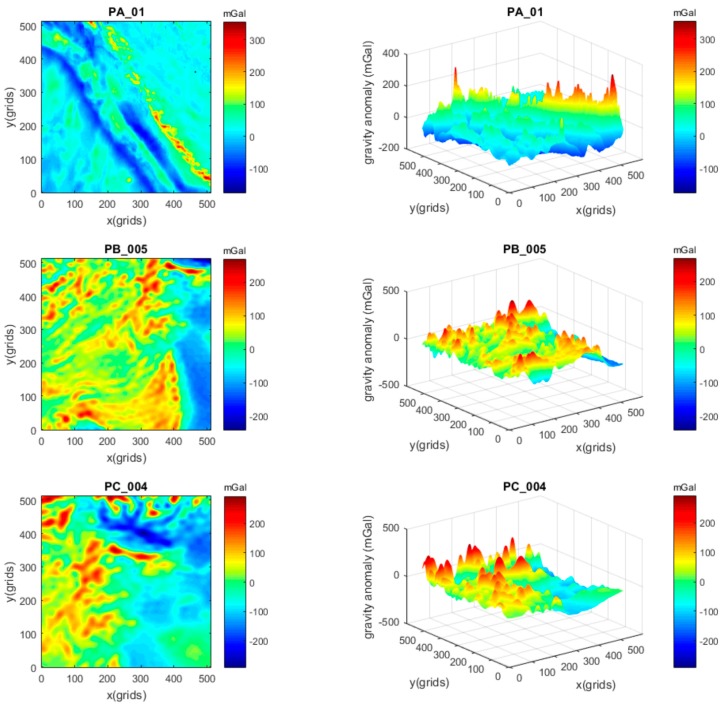
Gravity maps PA_01, PB_005, and PC_004.

At first, three gravity maps with different resolutions, but similar AGD values, were chosen to implement the simulation. The selection of similar AGD values was an attempt to reduce the impact of different gravity changes. The 2D and 3D views of the maps PA_01, PB_005, and PC_004 are demonstrated in Figure 3. The gravity maps PB_005 and PC_004 were constructed from 1′ × 1′ resolution maps located in different areas with an interpolation method. All three maps contain 512 × 512 grids, but the spatial resolutions in both *x* and *y* directions are 1′, 0.5′, and 0.4′, respectively.

The gravity values in the maps are gravity anomalies, and their statistical values are given in Table 1, where STD is the standard deviation, RMS is the root mean square. AGD is the average gravity difference of the map where the values of the three maps are in the range from 2.20 to 2.60 mGal/grid.

**Figure 4:**
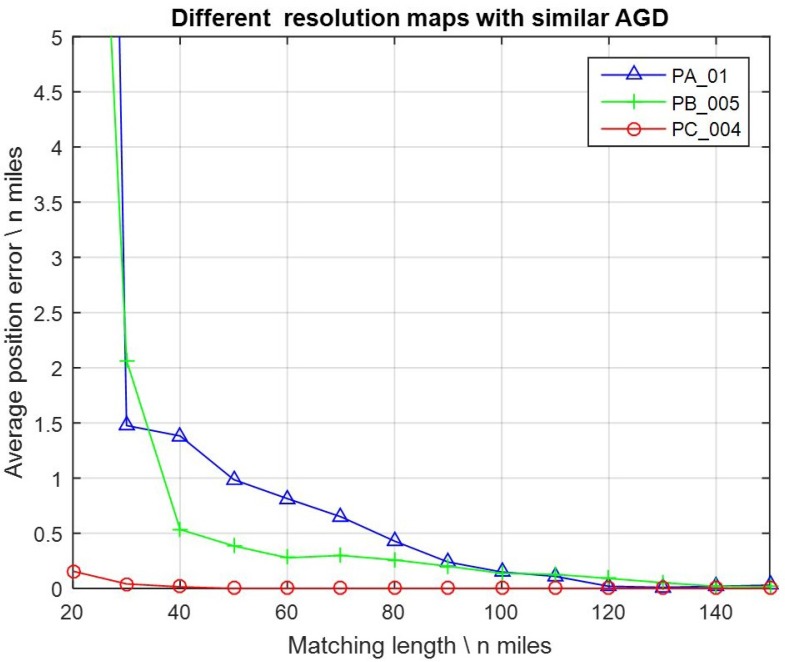
Average position errors with gravity maps PA_01, PB_005, and PC_004.

The FID values were assigned constantly with 3.001 mGal here for noise, by reference to the practical FID values between the altimeter-derived gravity DB and shipboard gravity. Matching lengths were assigned with 14 different values from 20, 30, 40, … to 150 nautical miles. For every map and every single value of the matching length, there was a 100 path tests. Then, the average position error can be calculated for each test. After a series of tests, average position errors can be collected and the results are presented as curves in Figure 4.

It can be seen that, in most cases, higher resolution maps (which means smaller resolution value) brought about obviously smaller average position error (which means more accurate). As the matching lengths were less than 40 nautical miles, it worked well with map PC_004, while the results were invalid with maps PA_01 and PB_005. On the other hand, with maps PA_01, PB_005, and PC_004, the average position accuracies achieved half a grid when the matching lengths reached 80, 60, and 20 nautical miles, respectively. Therefore, it can be verified that, in GMAN systems, higher resolution gravity DB or maps generally led to more accurate positioning results, and fewer measurement samples were needed.

#### 3.1.2. Different Resolution Maps in the Same Area

**Figure 5:**
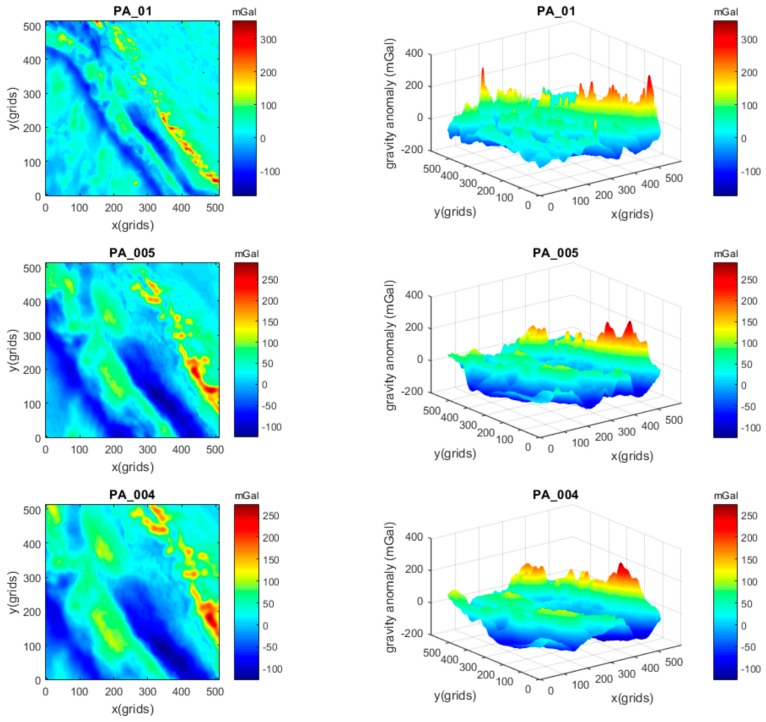
Gravity maps PA_01, PA_005, and PA_004.

By contrast, three gravity maps with different resolutions, but in the same area, were adopted. This selection was an attempt to reduce the impact of different areas. The 2D and 3D views of the maps PA_01, PA_005, and PA_004 are demonstrated in Figure 5. The maps PA_005 and PA_004 were obtained from PA_01 with an interpolation method. Similarly, all three maps contain 512 × 512 grids, but the spatial resolutions in both *x* and *y* directions are 1′, 0.5′, and 0.4′, respectively. The statistical values are given in Table 2. It can be seen that the AGD values of the maps became smaller when the resolutions increased, due to the interpolation for the same area.

**Figure 6:**
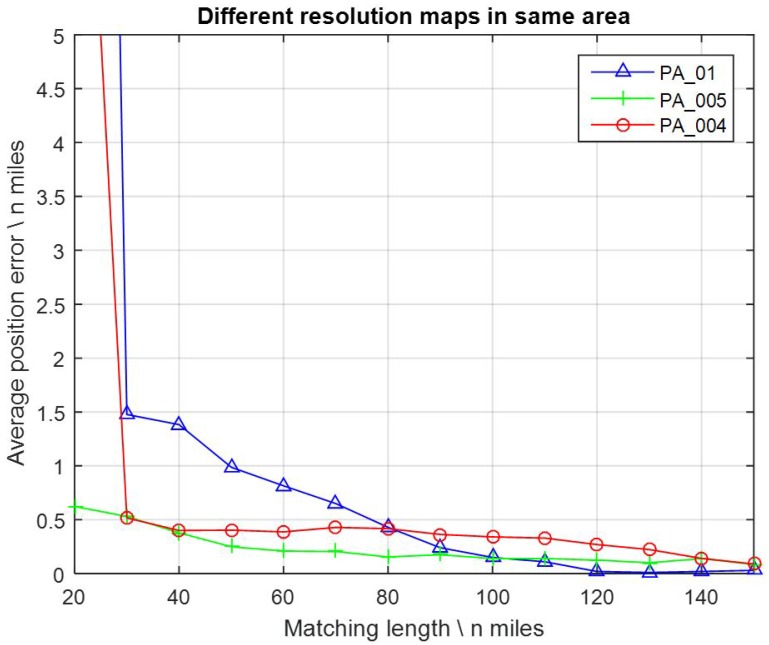
Average position errors with gravity maps PA_01, PA_005, and PA_004.

Similar to before, the FID values were assigned constantly with 3.001 mGal for noise. Matching lengths were assigned with 14 values from 20, 30, 40, … to 150 nautical miles. After a series of tests, the average position errors were calculated, as presented in Figure 6.

As the matching length was less than 80 nautical miles, the average position errors of PA_005 and PA_004 remained obviously smaller than PA_01. However, in other cases this advantage was not so clear, like the results before. This is probably since the smaller AGD of the map weakened the improvement of the higher resolution.

Furthermore, the comparison of results in Figure 4 and Figure 6 indicate that the effect of a higher resolution DB was significant in some areas where the AGD values were large enough (e.g., AGD > 2 mGal/grid), but was not that obvious in the areas where AGD values were small (e.g., AGD < 1.8 mGal/grid).

### 3.2. FID of Gravity Measurements

**Figure 7:**
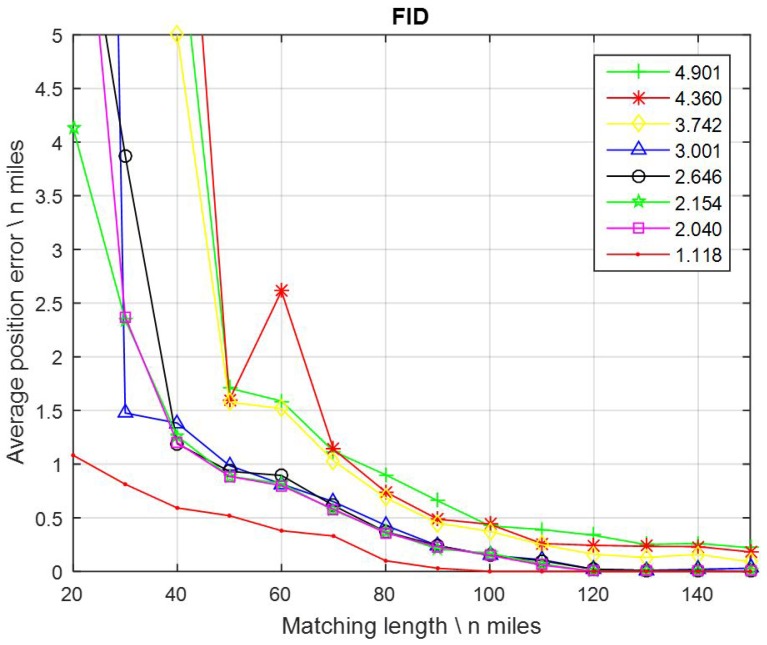
Average position errors of different FID values.

Gravity map PA_01, with a 1′ × 1′ resolution and AGD = 2.591 mGal/nautical mile, as shown before in Figure 3 and Figure 5, was used to implement the simulation. As mentioned before, currently the smallest FID values between the altimeter-derived gravity DB and shipboard gravity range from 1.6 to 3.6 mGal. In addition, most marine gravimeters in the world today have a dynamic measuring accuracy of 1 to 2 mGal. In consideration of these, the FID values were assigned with eight different numbers: 1.118, 2.040, 2.154, 2.646, 3.001, 3.742, 4.360, and 4.901 mGal. These values almost covered all possible accuracies in the present and for the next several years. Matching lengths were assigned with 14 values from 20, 30, 40, … to 150 nautical miles. After the tests, the average position errors were calculated and are displayed in Figure 7.

It can be seen that, with the same gravity DB, which means same resolution and AGD, different FID values led to totally different positioning results. Sometimes the average position errors obtained from some nearby FID values were also close to each other, for instance, FID = 4.901, 4.360, and 3.742, or FID = 3.001, 2.646, 2.154, and 2.040. Nevertheless, smaller FID values led to higher position accuracies, generally.

Additionally, the performance of positioning became better when FID < 1.118 and worse when FID > 3.001. By contrast, the FID of the gravity measurements had a relatively greater impact on positioning than DB resolution.

### 3.3. Number of Measurement Samples

**Figure 8:**
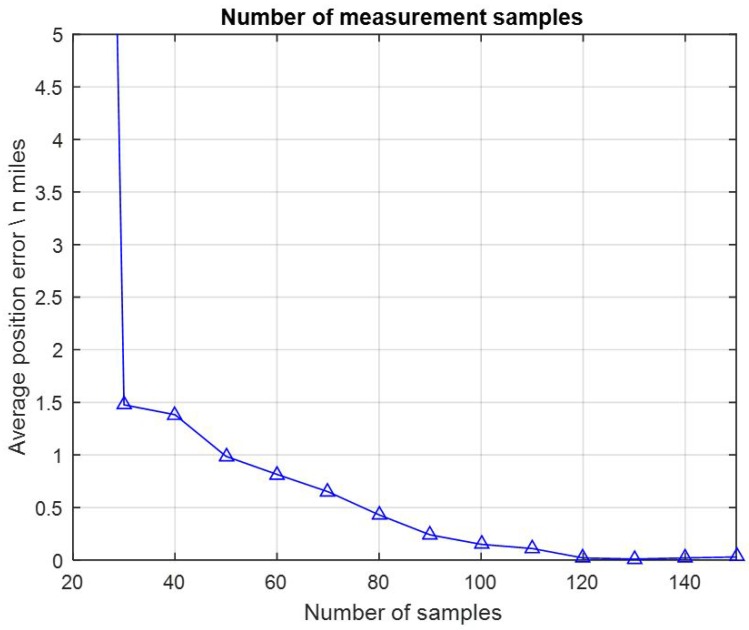
Average position errors with different numbers of measurement samples.

Actually, the influence that the number of measurement samples had can be seen from the results above in Section 3.1 and Section 3.2. One set of the results were separately shown in Figure 8. Gravity map PA_01, with a 1′ × 1′ resolution and AGD = 2.591 mGal/nautical mile, was employed, FID values were assigned with 3.001 mGal constantly. The number of measurement samples were assigned with 14 values from 20, 30, 40, … to 150. The average position errors with different numbers of measurement samples are presented in Figure 8.

In this test the average position errors reached a 0.5 nautical mile accuracy when the matching length was larger than 80 nautical miles. Obviously, it can be seen that the average position errors decreased all the time while the number of samples increased. Along with Section 3.1 and Section 3.2, these results were in good agreement with the theoretical prediction that better positioning performance could be gained with more measurement samples, which means more information.

### 3.4. Gravity Changes in Matching Area

**Figure 9:**
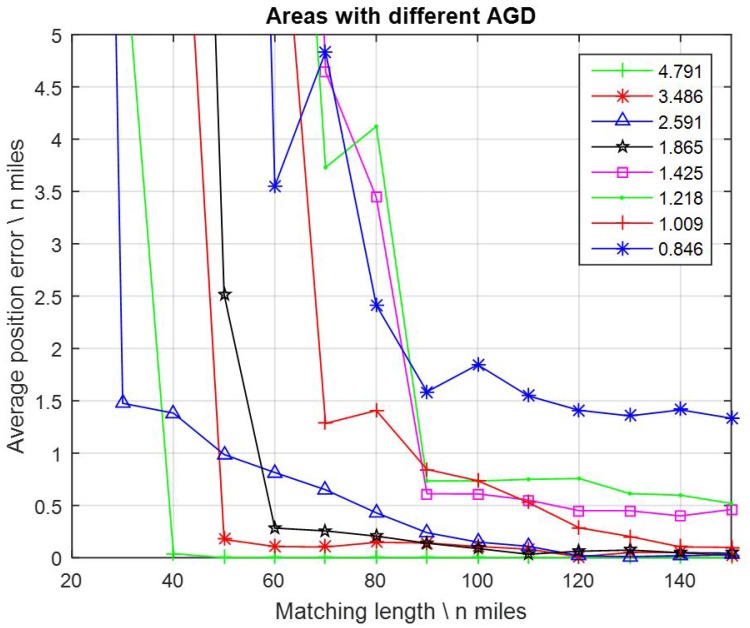
Average position errors in different areas.

Eight 1′ × 1′ resolution gravity maps located in different areas were chosen to the tests. The AGD values of these maps ranged from 0.846 to 4.791 mGal/nautical mile. FID values were assigned with 3.001 mGal constantly. Matching lengths were assigned with 14 values from 20, 30, 40, … to 150 nautical miles. The average position errors with maps of different AGD values are displayed in Figure 9.

It appears that different gravity changes in the matching area which were indicated by AGD values played a much more serious role than any other factors. Even not so strict, in most cases the average position errors decreased when AGD values increased.

In particular, with AGD values < 1.865 mGal/nautical mile, the average position errors became much larger, as well as the matching length needing to be at least 120 nautical miles to obtain a 0.5 nautical mile positioning accuracy. Especially for AGD = 0.846 mGal/n mile, the average position errors remained larger than 1.3 nautical miles, even when the matching length were enlarged to 150 nautical mile. By contrast, with AGD > 2.591 mGal/nautical mile, the average position errors became much smaller, and the matching length needed to be no more than 50 nautical miles to obtain a 0.5 nautical mile positioning accuracy.

Based on the results and discussions of the tests, a general equation was constructed to approximately describe the relationship between average position accuracy of GMAN and the four factors:

(5) $$ACCURACY=\frac{\alpha \cdot RES\times \beta \cdot FID}{\mu \cdot NOS\times \nu \cdot AGD}$$

where *RES* is the DB resolution, *NOS* is the number of measurement samples, and *α*, *β*, *μ*, *ν* are the control coefficients. It can be found that these four factors are intertwined, and the weakest link would limit GMAN performance despite improvements in the other areas.

## 4. Requirements Analysis for GMAN

In consideration of the current positioning accuracy of GMAN and other aided navigation methods, three typical accuracy targets were proposed here to analyze the system requirements: 1.0, 0.5, and 0.2 nautical miles. To achieve these accuracy targets, several different system requirements are listed in Table 3, based on the tests accomplished which are mentioned above.

If 1.0 nautical mile positioning accuracy was required, with a 1′ × 1′ resolution DB and a marine gravimeter of ~1 mGal measuring accuracy, and FID = 3.001 mGal, the matching length should be at least 50 nautical miles in a selected matching area which has an AGD = 2.591 mGal/nautical mile. When the measuring accuracy became worse, the FID value increased; for example, when FID = 4.360, the matching length should be extended to 70 nautical miles. Additionally, when the gravimeter accuracy can be maintained as FID = 3.001, but the matching areas with smaller AGD values were selected, longer matching lengths were required to reach the same positioning accuracy target. On the contrary, the matching length could be shortened if the gravimeter accuracy improved (smaller FID values), higher resolution gravity DBs were adopted, or matching areas with larger AGD values were selected. Similar laws can be concluded from the other two positioning accuracy targets.

Likewise, if higher positioning accuracies were demanded, the GMAN system requirements absolutely need to be improved, which means higher resolution gravity DBs, better gravimetry accuracy (smaller FID values), more measurement samples (longer matching lengths), or matching areas with rougher gravity changes (larger AGD values).

In practice sometimes the size of the matching area was confined, so the matching lengths were limited. If the matching lengths were restricted to no longer than 50 nautical miles, the following requirements should be satisfied.

1. For 1.0 nautical miles positioning accuracy (~1853 m)

FID ≤ 3.001 and AGD ≥ 2.591 with 1′ resolution DB, or FID ≤ 3.001 and AGD ≥ 2.220 with 0.5′, 0.4′, or 0.2′ resolution DB. This means that if the positioning accuracy target of the GMAN system was 1.0 nautical mile, just the existing 1 mGal accuracy gravimeter (FID ≤ 3.001), DB resolution (1′) and selected matching areas (AGD ≥ 2.591) were sufficient. Certainly some higher resolution DB (0.5′, 0.4′, or 0.2′) with selected matching areas (AGD ≥ 2.220) would be better.

2. For 0.5 nautical mile positioning accuracy (~927 m)

FID ≤ 1.118 or AGD ≥ 3.486 with 1′ resolution DB, or FID ≤ 3.001 and AGD ≥ 2.220 with 0.5′, 0.4′, or 0.2′ resolution DB. This means that if the positioning accuracy target was 0.5 nautical miles, the existing resolution DB (1′) should be combined with higher accuracy gravimeter (FID ≤ 1.118) or matching areas with the largest gravity changes (AGD ≥ 3.486). By comparison, higher resolution DB (0.5′, 0.4′, or 0.2′) could be combined with just the existing 1 mGal accuracy gravimeter (FID ≤ 3.001) and selected matching areas (AGD ≥ 2.220).

3. For 0.2 nautical mile positioning accuracy (~371 m)

FID ≤ 3.001 and AGD ≥ 3.486 with 1′ resolution DB, or FID ≤ 3.001 and AGD ≥ 2.220 with 0.4′ or 0.2′ resolution DB. This means that if the positioning accuracy target was 0.2 nautical miles, the existing resolution DB (1′) and 1 mGal accuracy gravimeter (FID ≤ 3.001) must be combined with the matching areas with the largest gravity changes (AGD ≥ 3.486). Additionally, higher resolution DB (0.4′ or 0.2′) and the 1 mGal accuracy gravimeter (FID ≤ 3.001) need to be combined with the selected matching areas (AGD ≥ 2.220).

The improvement of the gravity DB resolution in a large area and the reduction of measurement FID values depend on the development of satellite altimetry, marine gravimetry, data processing, and some other related technologies. However, the regional DB resolution can make some progress with shipboard gravimetry. The number of samples or matching lengths can be increased with more sailing time or further distance. High AGD values can be acquired through the selection of matching areas which have rough gravity changes. Based on the above analysis, at present the most effective and efficient approach to improve the positioning accuracy of GMAN is to select suitable matching areas with larger gravity changes which can be implemented with the gravity DB before sailing. Next, to obtain a higher resolution DB in regional areas where gravity changes roughly by shipboard gravimetry, or to obtain longer matching lengths by extending the sailing time/distance, are also feasible methods. Furthermore, the advancement of satellite altimetry and marine gravimetry would be significant benefits, and the GMAN positioning accuracy would be increase to a new level.

The most popular example of map-matching navigation systems is the terrain referenced navigation (TRN) system. The positioning accuracy of TRN has achieved 100 meters in magnitude in recent years, and several systems, like TERCOM and Sandia inertial terrain-aided navigation (SITAN), have already been utilized in airplanes, submarines, and missiles [31]. In the underwater environments, the performance of TRN would be seriously influenced by depth, temperature, and salinity of the water, and so on. Similarly, DB resolution, FID values, number of samples, and terrain changes in the matching area are also significant for TRN. By contrast, GMAN may not achieve TRN levels of accuracy, currently, but it is not emanative, not easily detected and interfered with, and provides passive, all-weather, and undeniable position updates with respectable accuracy.

## 5. Conclusions

In this paper, simulations were accomplished to evaluate the performance of gravity matching aided navigation (GMAN) under the influence of various factors, such as DB resolution, fitting degree of gravity measurements, number of samples in matching, and gravity changes in the matching area. A gridded marine gravity DB, which was calculated from satellite altimetry and shipborne surveys, was employed. Actual dynamic gravimetry accuracy and operating conditions were referenced to design the simulation parameters. The results verified that the improvement of DB resolution, gravimetry accuracy, number of measurement samples, or gravity changes in matching areas generally led to higher positioning accuracies. However, the effects of these factors were different and interrelated. Additionally, three typical positioning accuracy targets of GMAN were proposed, conditions to achieve these targets were concluded based on the analysis of several different system requirements.

The performance evaluation and requirements analysis in this paper provided several different approaches to improve the positioning accuracy of GMAN. It can be considered as a reference for actual marine experiments and the further development of related technologies. For focusing on the gravity matching process of GMAN, the four key factors were chosen in this paper. Moreover, various circumstances, including underwater, long period, large area, high-low latitudes, environment of vehicles, sea conditions, temperature, and salinity of the water, should also be taken into consideration in future marine experiments and performance evaluations.

## Figures and Tables

**Table 1:** Parameters of gravity maps PA_01, PB_005, and PC_004.

Maps	Grids	Spatial Resolution	Statistical Values of Gravity Anomalies (mGal)					AGD (mGal/grid)
			Max.	Min.	Mean	STD	RMS	
PA_01	512 × 512	1′ × 1′	353.436	−174.368	18.759	47.806	51.355	2.591
PB_005	512 × 512	0.5′ × 0.5′	267.227	−239.898	30.216	67.411	73.873	2.272
PC_004	512 × 512	0.4′ × 0.4′	290.747	−290.133	−17.835	87.062	88.870	2.232

**Table 2:** Parameters of gravity maps PA_01, PA_005, and PA_004.

Maps	Grids	Spatial Resolution	Statistical Values of Gravity Anomalies (mGal)					AGD (mGal/grid)
			Max.	Min.	Mean	STD	RMS	
PA_01	512 × 512	1′ × 1′	353.436	−174.368	18.759	47.806	51.355	2.591
PA_005	512 × 512	0.5′ × 0.5′	288.713	−124.454	16.158	55.560	57.862	1.701
PA_004	512 × 512	0.4′ × 0.4′	273.338	−124.439	18.487	56.732	59.668	1.504

**Table 3:** GMAN system requirements for typical positioning accuracy targets.

Typical Positioning Accuracy Targets (n mile)	Requirements				
	DB Resolution (n mile)	FID (mGal)	Number of Samples	Matching Length (n mile)	AGD of Matching Areas (mGal/grid)
1.0	1	4.360	70	70	2.591
	1	3.001	50	50	2.591
	1	2.040	40	40	2.591
	1	1.118	20	20	2.591
	0.5	3.001	70	35	2.272
	0.4	3.001	40	16	2.232
	0.2	3.001	60	12	2.220
	1	3.001	90	90	1.425
	1	3.001	60	60	1.865
	1	3.001	50	50	3.486
0.5	1	4.360	90	90	2.591
	1	3.001	80	80	2.591
	1	2.040	80	80	2.591
	1	1.118	50	50	2.591
	0.5	3.001	80	40	2.272
	0.4	3.001	50	20	2.232
	0.2	3.001	60	12	2.220
	1	3.001	120	120	1.425
	1	3.001	60	60	1.865
	1	3.001	50	50	3.486
0.2	1	4.360	150	150	2.591
	1	3.001	100	100	2.591
	1	2.040	90	90	2.591
	1	1.118	80	80	2.591
	0.5	3.001	180	90	2.272
	0.4	3.001	50	20	2.232
	0.2	3.001	90	18	2.220
	1	3.001	150+	150+	1.425
	1	3.001	80	80	1.865
	1	3.001	50	50	3.486
